# Dose standardization for transcranial electrical stimulation: an accessible approach

**DOI:** 10.1038/s41598-025-25649-2

**Published:** 2025-11-25

**Authors:** Jake Toth, Méadhbh Brosnan, Rory-Jay King, Boyan Ivanov, Mahnaz Arvaneh

**Affiliations:** 1 School of Electrical and Electronic Engineering, University of Sheffield, Sheffield, S1 3JD UK; 2 Insigneo Institute for In Silico Medicine, University of Sheffield, Sheffield, S10 2TN UK; 3 Neuroscience Institute, University of Sheffield, Sheffield, S10 2HQ UK; 4 School of Psychology, University College Dublin, Dublin 4, Ireland; 5 Department of Experimental Psychology, University of Oxford, Oxford, OX2 6GG UK; 6 Department of Psychiatry, Oxford Centre for Human Brain Activity, Wellcome Centre for Integrative Neuroimaging, Oxford, OX3 7JX UK; 7 Turner Institute for Brain and Mental Health, School of Psychological Sciences, Monash University, Melbourne, VIC 3800 Australia; 8 Present Address: Institute of Biomedical Engineering, University of Oxford, Oxford, OX3 7DQ UK

**Keywords:** Biotechnology, Biophysical models

## Abstract

Transcranial electrical stimulation (tES) is a widely used non-invasive brain stimulation technique. However, due to high inter-individual variability in the induced electric fields (E-fields), a fixed stimulation current delivers an inconsistent dose. We developed a dose standardization method without the requirement of participant-specific structural imaging and E-field modeling. Robust multiple linear regression models were trained to predict peak E-field strengths across 10 electrode montages and 418 healthy adults. These regression models predicted peak E-field strengths in unseen participants from accessible demographic and morphological parameters. Estimated peak E-field strength values were subsequently used to standardize tES dosages across our population. Additionally, we developed montage-agnostic models which incorporated inter-electrode distances for each participant. Compared to fixed dosing, our approach significantly reduced peak E-field strength variation for conventional montages, though results were inconsistent for high-definition (HD) montages. Models trained on specific montages accounted for 43% of peak E-field strength variability in conventional montages and 21% in HD montages on average. Our montage-agnostic models accounted for 36% and 13% of the average peak E-field strength variability for conventional and HD montages, respectively. These results have been validated across a large dataset, demonstrating robust performance against unseen data, a significant advancement over current approaches.

## Introduction

Transcranial electrical stimulation (tES) is a non-invasive brain stimulation method that influences brain activity primarily by modulating neuronal membrane potentials^1^. This is achieved by applying a weak electrical current (typically 1-4 mA) through one or more electrodes placed on the scalp. The potential applications of tES are broad, ranging from treating depression^2^ and long COVID^3^ to mild cognitive impairment^4^.

However, the apparent efficacy of tES has been hampered by the variability in its effects^5^. A potential source of this variability lies in the heterogeneity of the electric fields (E-fields) generated within the brain across individuals, even with identical stimulation parameters^6^. For instance, the strength of the E-field generated in the motor cortex can vary by up to 100% between individuals given a fixed stimulation dose^7^. Increasing evidence suggests that E-field strength may be directly linked to behavioral changes (e.g.,^8–10^), highlighting the importance of addressing this inter-individual variability to improve tES outcomes^11,12^.

Individualized E-field simulations, which use participant-specific structural MRI scans and finite element methods, offer a promising solution for estimating individualized tES dosing. This approach has been validated through intracranial recordings^13^, cerebral blood flow measurements^14^, and associations with neurotransmitter changes^15^. However, the requirement for MRI scans and specialized expertise presents a significant barrier to widespread adoption, undermining the cost-effectiveness and accessibility of tES^11^.

Inter-individual E-field variability can arise due to variations in anatomic factors, including bone density^16^, cerebrospinal fluid (CSF) thickness^6^, and head circumference^17^. E-fields have also been found to vary with demographic factors such as sex^18^, age^19,20^, and the inter-electrode distance^21^. Here, we ask whether, by taking some of this variability into account, we can estimate individualized doses to standardize the dose received across our population. (See^17^ for a previous demonstration of the feasibility of such an approach using head circumference in 47 participants).

Here, we trained a series of robust multiple linear regression (MLR) models on a large E-field dataset of 418 adults to estimate participant-specific dosing across 10 commonly used tES montages. We trained the models using independent variables that are straightforward to obtain in most laboratory and clinical environments, with the notable exclusion of costly neuroimaging metrics. These variables include age, gender, head circumference, cephalic index (head width to length ratio), body mass index, and inter-electrode distance.

More specifically, first, we tested the independent associations between these variables and MRI-informed peak E-field strengths across the 10 montages using Spearman’s rank-order partial correlations. We then trained and validated montage-specific robust MLR models for each montage using 5-fold cross-validation to estimate the required dose for unseen participants. The estimated peak E-fields from our models were compared to those obtained using MRI-informed E-field simulations, using normalized root-mean-squared error and adjusted R^2^.

To demonstrate the scalability of our approach to novel montages, we proposed montage-agnostic dose estimation models. This was achieved using two types of regression models: one incorporating inter-electrode distance as a linear term and another including inter-electrode distance as both linear and quadratic terms. The proposed montage-agnostic models were evaluated on previously unseen montages and unseen participants.

In summary, this paper presents a simple, scalable approach toward standardizing tES dosages without participant-specific MRI scans. It also introduces a method to generalize individualized dose estimation to novel, unseen montages.

## Results

### Peak E-field strength variation with age, gender, and head morphology

To ensure the artifact rejection and outlier removal processes did not introduce selection bias, we compared the demographics of the original dataset (N = 653) with the reduced dataset post-processing (N = 418). We used a Chi-squared test for the participants’ gender, the pairwise Kolmogorov-Smirnov test for age, and the Mann-Whitney U test for BMI distribution, excluding participants whose BMI was not calculable from the original dataset. These tests indicated no statistically significant differences ($p>.05$).

Pearson’s linear correlation between head circumference and MRI-informed peak E-field strength confirmed significant negative correlations across all montages ($p<.01$). Additionally, Spearman’s Rho correlation analysis indicated significant negative correlations between age and peak E-field strength ($p<.001$) and BMI and peak E-field strength ($p<.05$). The cephalic index was negatively correlated with peak E-field strength in all conventional montages ($p<.001$), but only in the HD montage with the anode at PO8. Additionally, independent t-tests revealed significant peak E-field differences between genders in 5 out of the 10 montages. A detailed table of results can be found in the supplementary materials.

**Fig. 1:**
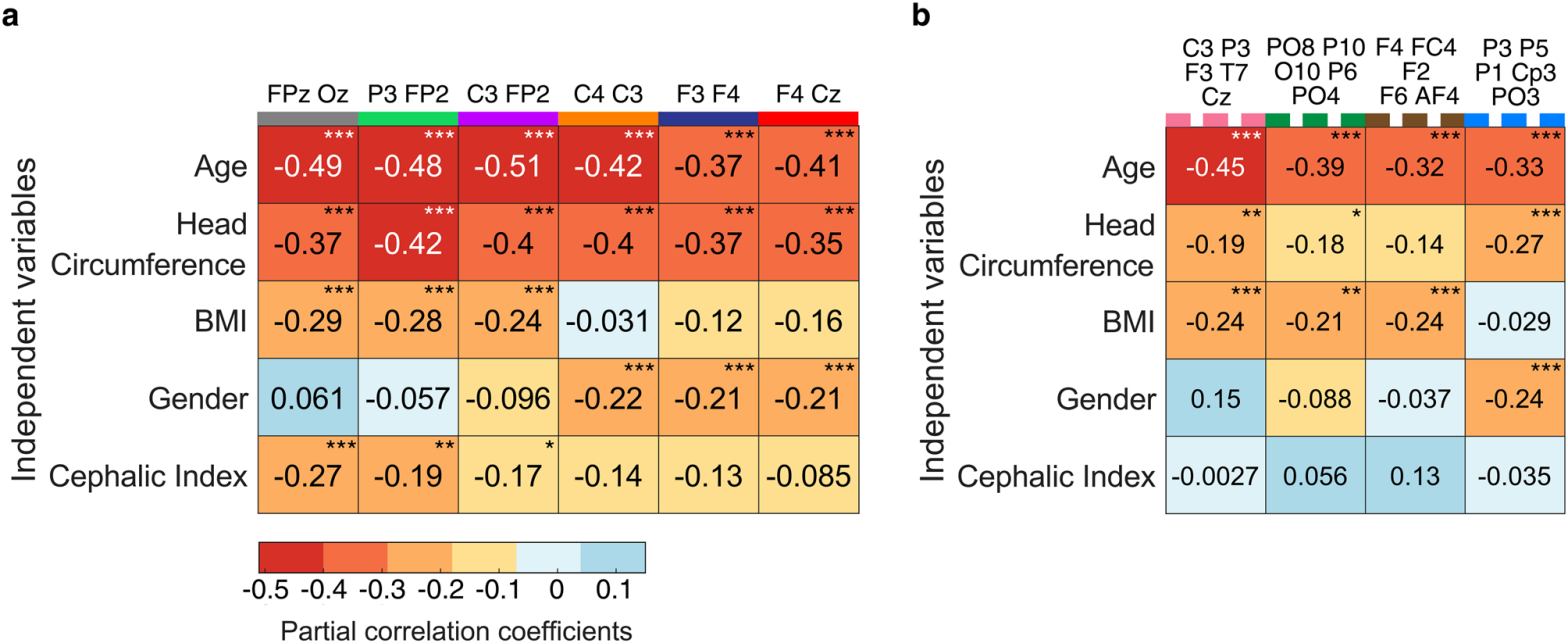
Relationship between anatomical and demographic variables and peak E-field strength. Partial Spearman’s rank-order correlations ($r_{s}$) are shown for (**a**) conventional montages and (**b**) high-definition (HD) montages. Each coefficient was computed while controlling for all other independent variables. P-values were Bonferroni adjusted for multiple comparisons $^{\text {***}}p <.001$, $^{\text {**}}p<.01$ and $^{\text {*}}p<.05$.

Thereafter, we performed Spearman’s rank-order partial correlations between each variable and the peak E-field strength, controlling for all other variables as shown in Figure 1. We observed that age is the most prominent factor ($p<.001$ across all montages), followed by head circumference ($p<.001$ across all conventional montages). Finally, we observed a weak correlation between the cephalic index and peak E-field strength, particularly in anterior-posterior montages.

**Fig. 2:**
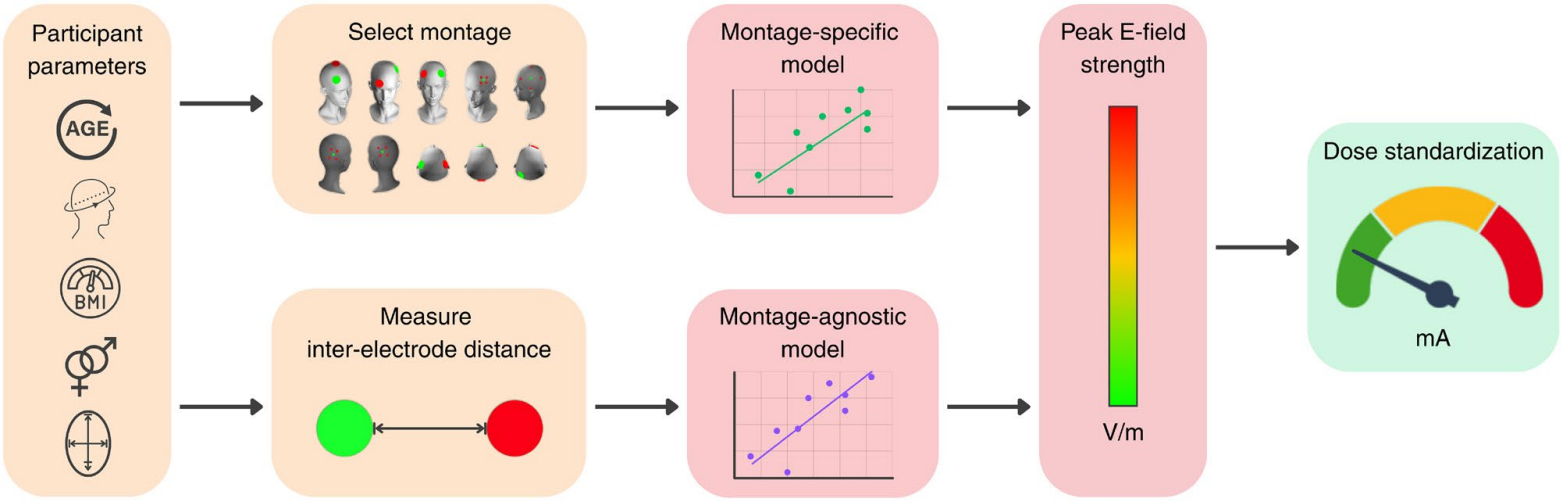
Illustration of the workflow for montage-specific and montage-agnostic dose standardization. For all models, accessible parameters are measured, including age, gender, head circumference, cephalic index and BMI. Both montage-specific and montage-agnostic models assume that +1 mA of current is applied to the anode and $-$-1mA to the cathode. In montage-specific models, these parameters are entered into multiple linear regression models to estimate the peak E-field strength for each individual. Montage-agnostic models require an additional input: the inter-electrode distance, which is incorporated into the respective models for conventional and HD montages. The estimated peak E-field values can then used to standardize dosage across individuals.

We compared the peak E-field strengths predicted by our models against values obtained directly from the MRI scans across 10 montages and 5-fold test sets. Figure 5 illustrates the proportion of variance in the peak E-field strength explained by our models, measured by the adjusted R^2^ on test sets. Models trained on conventional montages had a mean adjusted R^2^ of 0.43, and those trained on HD montages had a mean adjusted R^2^ of 0.21, as shown in Fig. 5. Anterior-posterior conventional montages (anode: FPZ, cathode: Oz), (anode: P3, cathode: FP2), (anode: C3, cathode: FP2) had the highest performance, likely related to the significant partial correlations with the cephalic index. HD montages with the closest inter-electrode distances (anode: P3) and (anode: F4) performed worse. Our dose standardization approach, illustrated in Fig. 2, reduced the standard deviation of the peak E-field strengths across the population. On average, the standard deviation was reduced by 25.8% and 13.6% for conventional and HD montages respectively, compared to a fixed dose approach.

### Montage-specific dose standardization

**Fig. 3:**
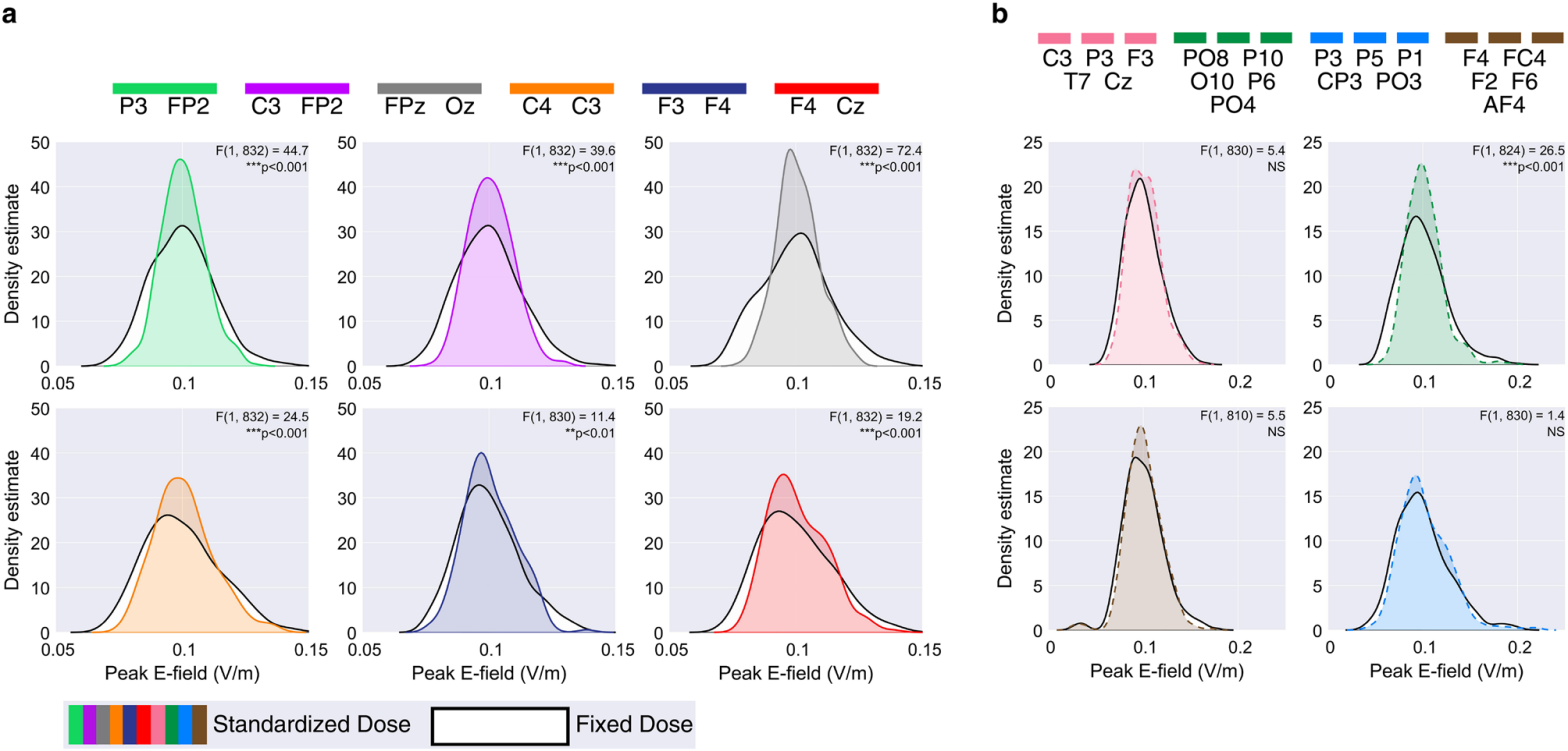
Effect of dose standardization on peak E-field strength distributions. Peak E-field distributions from the standardized dosing approach (color) are compared against a fixed dose approach (black and white). Results are shown for (**a**) conventional montages and (**b**) high-definition (HD) montages. Levene’s tests were used to assess for differences in variance between the two approaches. P-values were Bonferroni adjusted for multiple comparisons, NS denotes a non-significant result.

Figure 3 compares the distributions of peak E-field strengths induced by a 1mA fixed-dose, scaled to have a mean of 0.1V/m, with those induced by standardized dosages determined by our models. Notably, for all conventional montages, we observed a significant reduction in peak E-field variability ($p<.01$) when applying our proposed standardized dosages. In HD montages, while standardized dosages generally reduced peak E-field strength variability, only one montage (anode at PO8) showed a significant reduction ($p<.001$).

### Montage-agnostic dose standardization

**Fig. 4:**
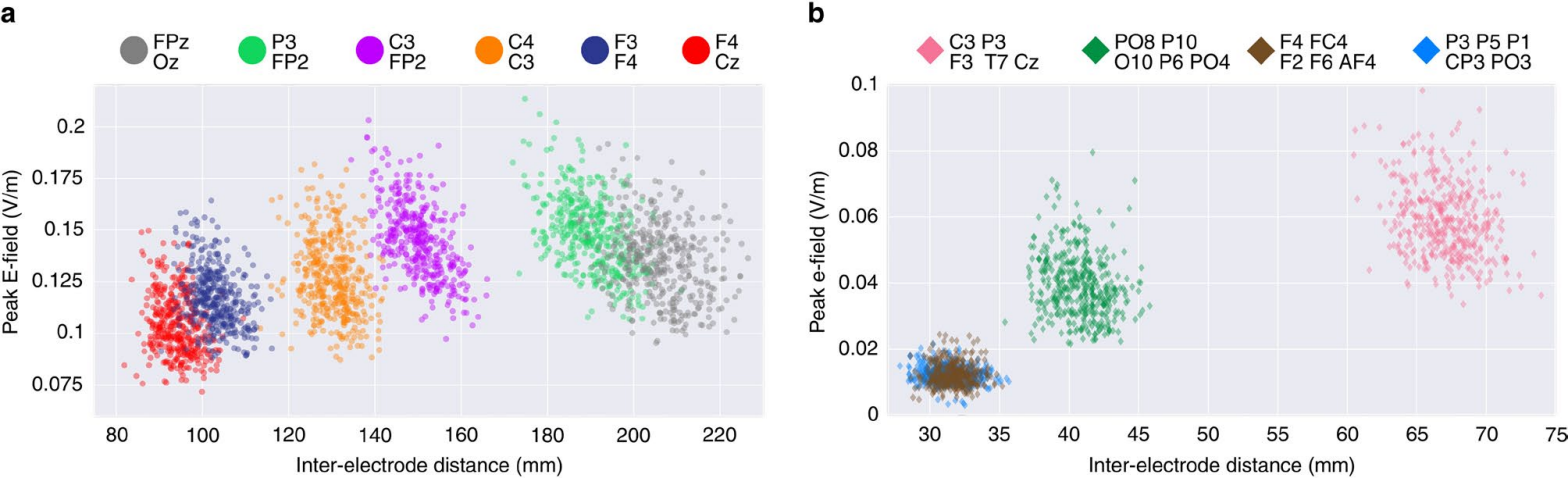
The relationship between the peak E-field strength and inter-electrode distances across (**a**) conventional montages and (**b**) high-definition (HD) montages.

In montage-agnostic models, we included inter-electrode distance as an additional input alongside the features used in montage-specific models. The relationship between inter-electrode distance and the peak E-field is shown in Fig. 4.

To develop montage-agnostic models, in the first instance, we used robust MLR. These models accounted for 35% of the peak E-field strength variability in conventional montages and 13% of the peak E-field strength variability in HD montages. Subsequently, in an attempt to account for the non-linear relationship between inter-electrode distance and peak E-field strength, we integrated a quadratic term. These models performed comparably to the linear models, explaining 36% and 11% of the variance in conventional and HD montages, respectively. Results tables for all models can be found in the supplementary materials.

**Fig. 5:**
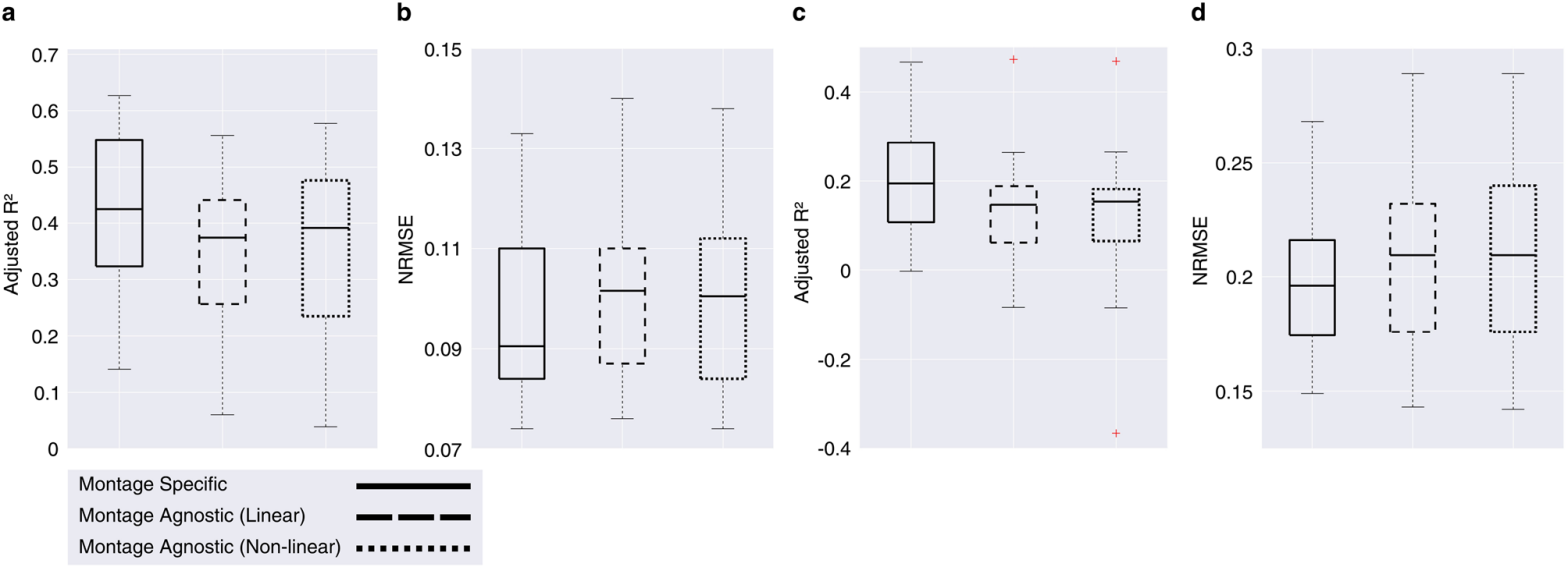
Performance of models in predicting peak E-field strength on test sets, evaluated against MRI-informed simulations. Model performance is shown for conventional montages in terms of **(a)** adjusted R^2^ and **(b)** normalised root-mean squared error (NRMSE). Performance for high-definition (HD) montages are shown in terms of **(c)** adjusted R^2^ and **(d)** NRMSE. The legend defines the three model types compared: montage-specific, linear montage-agnostic, and non-linear montage-agnostic. Box plots display the median (horizontal line), interquartile range (box), and data range (whiskers). Outliers are shown as red + markers.

The performance of the linear and non-linear models in terms of the adjusted R^2^ as well as NRMSE is shown in Fig. 5. Peak E-field variability between fixed-dose stimulation and standardized dosing is compared. Overall, montage-specific models explained more of the peak E-field variability than montage-agnostic models. Furthermore, the introduction of the quadratic term did not yield an appreciable improvement in performance in our dataset.

## Discussion

In this study we presented an accessible approach to tES dose standardization without requiring participant-specific MRI scans and extensive E-field modeling. We achieved this by incorporating a wide array of predictors, including age, gender, head circumference, cephalic index, BMI, and inter-electrode distance to estimate peak E-field strength using robust MLR models. Our models were developed and validated using a large dataset, comprising N=4,161 total simulations across 10 montages. Crucially, we validated these models against unseen test data. On average, montage-specific models accounted for 43% of peak E-field strength variability in conventional montages and 21% in HD montages. Montage-agnostic models accounted for an average of 36% and 13% of this variability in conventional and HD montages, respectively.

Age, followed by head circumference were the strongest predictors of peak E-field strength variability, according to Spearman’s partial regression analyses. Gender alone often showed a statistically significant influence on E-field strength. While peak E-field strength appeared higher in females than males, consistent with previous findings^22^, interpreting gender-related results requires caution due to the assumption of uniform resistivity across the skull. Local variations in skull conductivity may impact results across the genders^16^. Notably, we did observe a correlation between BMI and peak E-field strength in some montages, contrary to previous findings by Truong et al.^23^. However, in contrast to this work we did not segment fat tissue, likely leading to fat being misinterpreted as skin. BMI was also set to the population median (24.05 kg/m^2^) in 54 of the 418 participants which may have introduced bias.

Montage-specific models for HD montages do not appear to perform as well as those for conventional montages. However, caution should be exercised when making this direct comparison. The 95th percentile of the E-field magnitude within grey matter values was used as the measure of the peak E-field strength, which is consistent in volume regardless of the montage used. As a result, our measure of peak E-field strength did not account for the additional focality of HD montages, potentially making direct comparisons between the two montage types misleading. A region of interest (ROI) based approach has been utilized in previous works analysing montages with differing focalities^17,24^. A head circumference informed approach found that models trained on conventional montages performed better than those trained on HD montages^17^, which is consistent with the findings in work. This is to be expected, as HD montages have previously been reported to induce more variable E-fields compared to conventional montages^25^.

Montage-specific models outperformed montage-agnostic models in explaining peak E-field strength variability. We explored both linear and non-linear models when incorporating inter-electrode distance. The non-linear relationship between inter-electrode distance and peak E-field strength observed in our study has been noted in previous work^24^. However, we did not observe a notable difference in performance between the linear and non-linear montage-agnostic models, likely due to the small number of unique montages. For instance, in the case of HD montages, only three montages were used at any one time for training. Thus, further research incorporating additional montages may better capture a non-linear relationship between inter-electrode distance and peak E-field strength. Additionally, factors such as electrode size and shape must also be considered to ensure montage-agnostic dose estimation is universally applicable to novel montages.

The generalizability and safe usage of our dose standardization approach are constrained by the population of participants and the predictors used in the models. The effects of ethnicity and race were not considered. Participants were excluded from the CamCAN dataset for a variety of reasons, including cognitive impairment, MR safety and comfort, and serious head injury, among others^26^. Indications such as stroke^27^ and psychosis^28^ alter E-fields produced by transcranial electrical stimulation. As does the existence of skull defects, skull plates^29^ and burr holes in the skull^30^, all of which were not considered in this study.

Future work could improve model performance and applicability by addressing simulation inaccuracies, extending the analysis beyond peak E-field strength to more complex E-field measures, and incorporating additional participant-specific measures. Indeed, our supplementary analysis indicated that age was the strongest predictor of focality, which likely reflects underlying grey matter atrophy associated with aging^31^. Further analysis could be given to the spatial distribution and direction of the E-fields relative to the target neuron population^11^. Furthermore, while the 95th percentile of the E-field is broadly indicative of the dose received, metrics that incorporate multiple peak regions or an ROI based analysis may offer a more representative measure of effective dose. In this study, each tissue was given the same conductivity value in all participants. However, representative distributions of tissue conductivity across the population may produce more realistic E-fields^32^.

Future work could also incorporate more comprehensive E-field simulations through multi-layer skull models^33^, fat tissue segmentation^23^, anisotropic tissue conductivity^34^ and consideration for the impacts of microscopic features^35^. However, the application of increasingly complex techniques that may require additional imaging, compute, and expertise provide diminishing returns, particularly when these models are used to extract macro-scale E-field measures^36^.

The imaging techniques used to inform the E-field models could also be improved. For instance, the participant’s posture in the MRI scanner impacts the E-fields generated^37^ and it should therefore be representative of their posture during stimulation.

In addition to the aforementioned demographic and morphological data, other accessible parameters could also be used to inform dose standardization. These parameters would be collected through accessible imaging techniques such as electrical impedance tomography^38^, full-waveform inversion ultrasound imaging^39^ or resting state electroencephalography^40^. Additionally, generative modeling of structural MRI scans conditioned on participant-specific information^41^ could also be used to improve the E-field estimation.

Achieving consistent E-fields across participants through individualized dosing presents a fundamental challenge for tES research. Although participant-specific MRI scans and E-field simulations provide a precise solution, their high cost and limited accessibility hinder widespread use. This paper addressed these constraints by developing robust multiple regression models that could accurately predict peak E-field strength using easily accessible demographic data, head morphology, and inter-electrode parameters. Future experimental validation will be crucial to understanding the impact of our individualized dosing on tES efficacy and treatment outcomes.

## Methods

### Participants and magnetic resonance imaging

Structural MRI scans from the CamCAN repository (available at https://www.mrc-cbu.cam.ac.uk/datasets/camcan/)^26,42^ were used in this analysis. These scans were acquired with a 3T Siemens TIM Trio scanner with a 32-channel head coil at the Medical Research Council Cognition and Brain Science Unit in Cambridge UK. T1w scans were acquired using MPRAGE sequence with the following parameters: TR = 2250 ms, TE = 2.99 ms, TI = 900 ms, FA = 9$\circ$, field of view (FOV) = 256mm x 240mm x 192mm, voxel size = 1.0mm x 1.0mm x 1.0mm and GRAPPA acceleration factor = 2, TA = 4 min 32 s. T2w scans were acquired with a SPACE sequence with the following parameters: TR = 2800 ms, TE = 408 ms, flip angle = 9$\circ$, field of view = 256mm x 256mm x 192mm, voxel size = 1.0mm x 1.0mm x 1.0mm, GRAPPA acceleration factor = 2. After processing, the final cohort comprised 418 participants (202 male, 216 female), with a median age of 52 years (range: 18–87).

### Montage selection

**Fig. 6:**
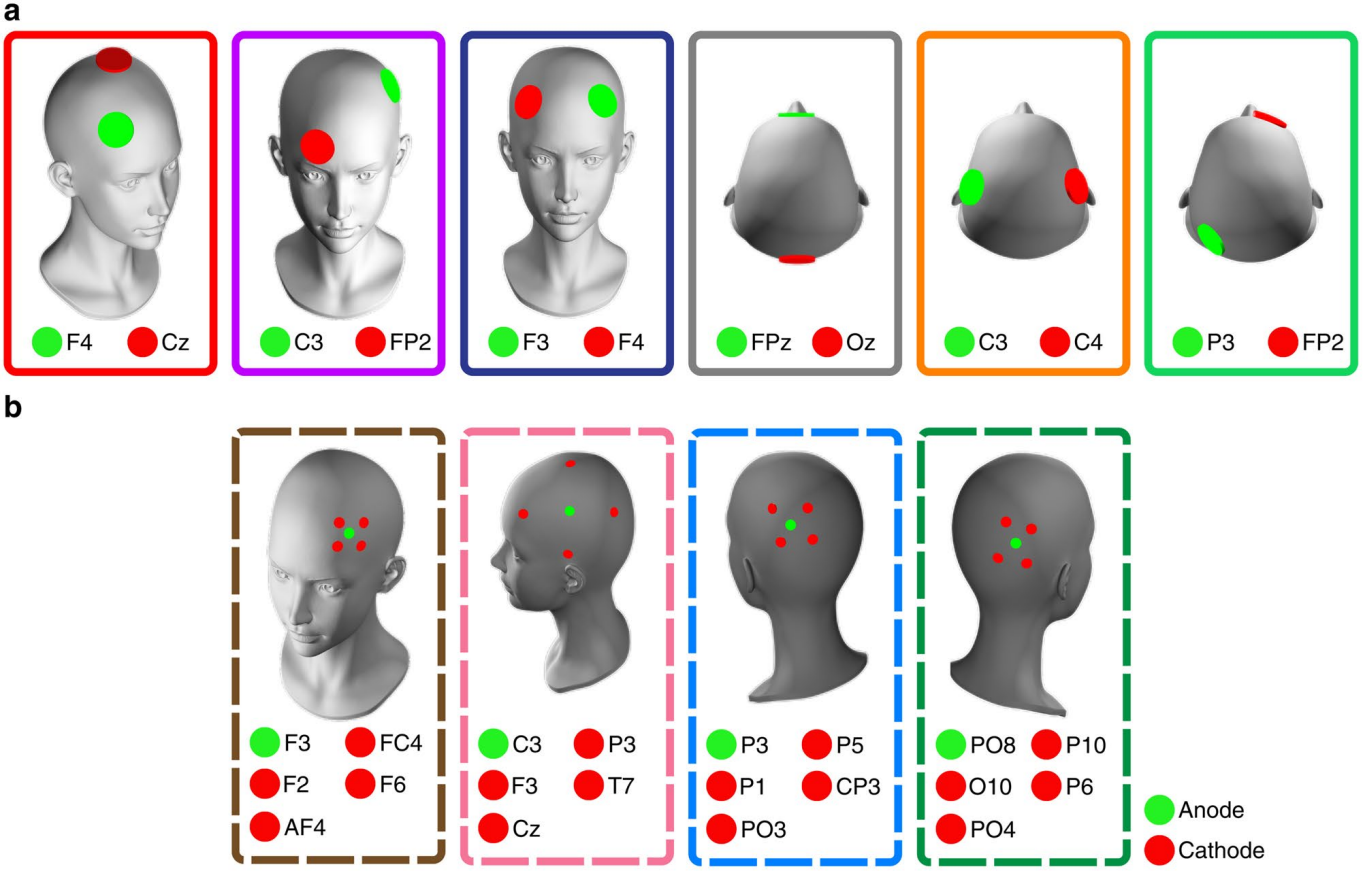
Electrode positions for the 10 transcranial electrical stimulation (tES) montages evaluated. The figure shows **(a)** the six conventional montages, where the anode (green) supplied +1mA and the cathode (red) was set to $-$1mA, and **(b)** the four high-definition (HD) montages, where a central anode (+1mA) was surrounded by four cathodes, each set to $-0.25$ mA.

Electrode positions were selected on the basis of their positional variation on the head and their use in previous works. At low stimulation intensities and frequencies often used in tES, the relationship between the applied current and the resulting E-field strength is linear^13^. Consequently, the E-field generated at a single reference intensity can be directly scaled to predict the E-field strength at other current values. All simulations were therefore based on a reference current of +1mA applied to the anode. In the case of conventional montages with a single cathode this +1mA anode corresponded to a cathode set to $-$1mA. Conventional montage positions are fronto-central (anode: F4, cathode: Cz)^43^, centro-frontal (anode: C3, cathode: FP2)^44^, cross-hemisphere frontal (anode: F3, cathode: F4)^45^, parietal-frontal (anode: P3, cathode: FP2)^46^, cross-hemisphere central (anode: C3, cathode: C4)^47^ and midline fronto-occipital (anode: FPz, cathode: Oz)^48^. HD montages included one central anode set to 1mA surrounded by four return electrodes set to $-$0.25mA. The HD montages were positioned as follows: frontal (anode: F4, cathodes: F2, AF4, F6, FC4)^49^, central (anode: C3, cathodes: P3, Cz, T7, F3)^50^, parietal (anode: P3, cathodes: P1, CP3, P5, PO3)^51^ and parietal-occipital (anode: PO8, cathodes: P6, PO4, P10, O10)^52^. All montages are visualized in Fig. 6.

### Tissue segmentation and manual curation

E-field simulations were performed using Realistic vOlumetric Approach-based Simulator for Transcranial electric stimulation (ROAST) version 3.0, an open-source, automated, E-field simulation pipeline^53^ in MATLAB (2021a). As part of the ROAST pipeline, all structural MRI scans were segmented using SPM12^54^. To produce more accurate meshing than using T1w scans alone^55^, both T1w and T2w MRI scans from all of the 653 participants were segmented into five different tissue types (white matter, grey matter, bone, skin and air). After which, all 653 segmentation maps were manually inspected for imperfections such as scalp contact with the CSF and incomplete eye segmentation. At this stage 200 participants were removed due to low-quality segmentation maps and 254 were manually adjusted to correct for minor errors. 199 segmentation maps were retained without corrections, resulting in a cohort of 453 participants for subsequent analysis.

### Electric field simulations

Following segmentation, electrodes were placed according to the respective montage being simulated. Conventional montages used electrodes with 28mm radii, while HD montages used electrodes with 10mm radii. Both types of electrodes were 1mm thick. Head and electrode meshes were generated with the MATLAB toolbox iso2mesh^56^. Finally, the finite element methods solver, getDP^57^, was used to solve the underlying Laplacian equation^58^. Isotropic electrical conductivities were assigned as follows; white matter $0.126$ S/m, grey matter 0.276 S/m, bone $0.01$ S/m, skin 0.465 S/m, air $2.5\times 10^{-14}$ S/m, gel $0.3$ S/m and electrode $5.9\times 10^7$ S/m. The conductivity value of 0.85 S/m was used for CSF to account for the meninges^59^. In this work, the strength of the E-field was considered, and the E-field direction was discarded.

### Electric field registration to MNI152 space

**Fig. 7:**
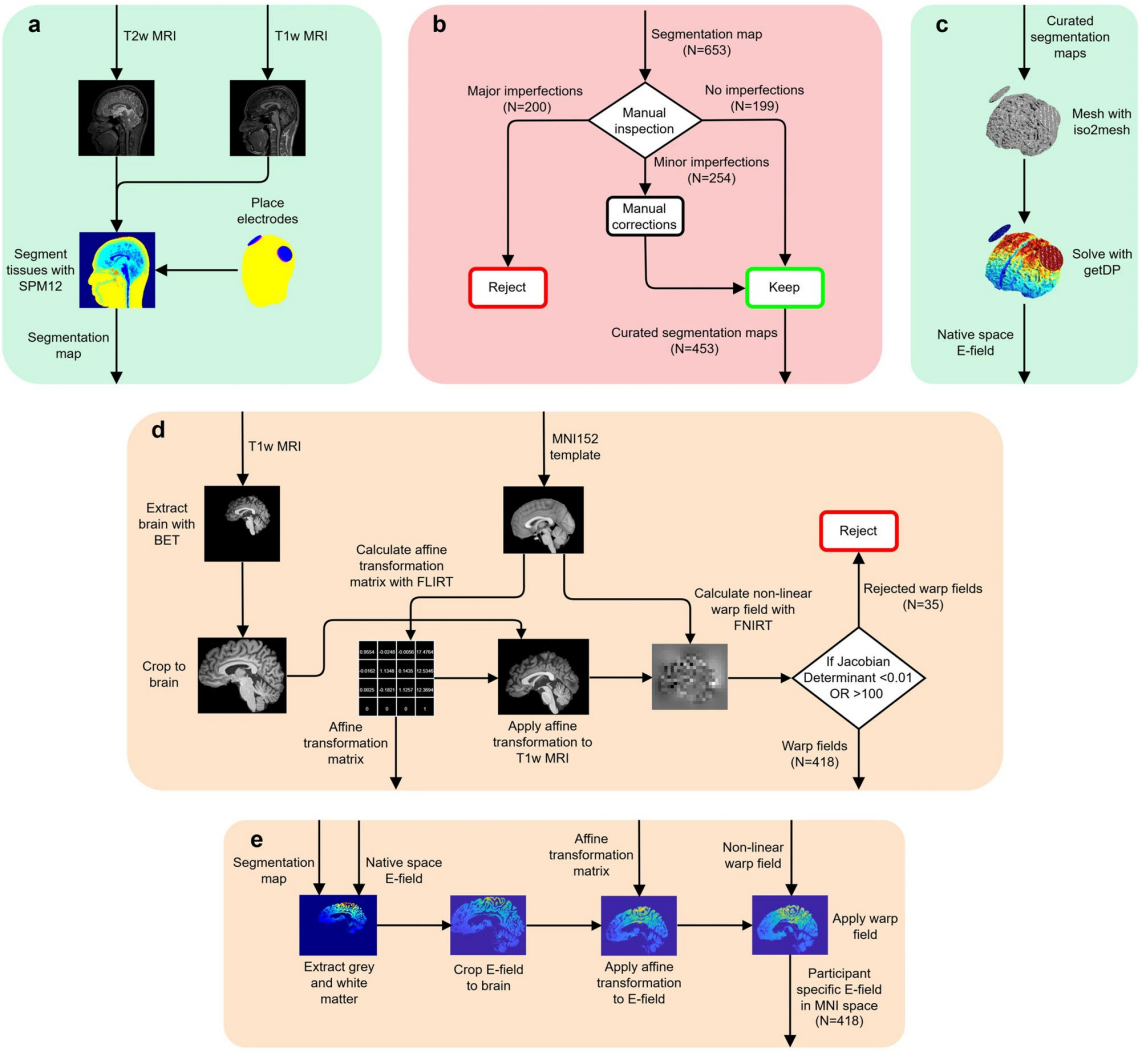
Processing pipeline for simulating the E-fields generated by transcranial electrical stimulation (tES) in MNI152 space from participant-specific structural MRIs. This process includes: (**a**) the generation of segmentation maps from T1w and T2w MRI scans performed using SPM12 within the ROAST pipeline. (**b**) Manual inspection and curation of segmentation maps. (**c**) Mesh generation from the curated segmentation maps using iso2mesh and the computation of native space E-fields using getDP as part of the ROAST pipeline. (**d**) The creation of affine transformation matrices and non-linear warp fields to transform native space MRI scans to MNI152 space using FSL tools (BET, FLIRT and FNIRT). (**e**) The application of these transformations to native space E-fields producing MNI152 space E-fields.

E-field results were transformed to the MNI152 6th generation reference space^60^. The complete pipeline is illustrated in Figure 7. These transformations used FSL version 6.0 (http://fsl.fmrib.ox.ac.uk/fsl/)^61^. Brain extraction (BET), followed by linear transformations (FLIRT)^62^ and non-linear transformations (FNIRT)^63^, were applied to the T1w MRI scans. 35 participants were excluded at this stage due to the Jacobian determinant of the FNIRT warp fields lying outside of a realistic range of values between 0.01 - 100^64^. The affine transformation matrices and warp fields were subsequently applied to E-field voxels corresponding to the grey matter in the segmentation maps used in the E-field simulation pipeline.

The peak E-field strength, defined as the 95th percentile of the E-field strength within grey matter voxels was extracted. The 95th percentile, rather than the peak voxel, was used to avoid partial volume effects. To ensure data quality, we removed extreme outliers from our dataset. Outliers were defined as peak E-field strength values that fell outside three times the interquartile range (IQR) for each montage. These extreme outliers may have been caused by the non-deterministic nature of mesh generation, which can cause local defects. Of the 4,180 E-fields generated across all 10 montages, 19 were rejected, resulting in the final dataset of 4,161 E-fields.

In addition to E-field strength analysis on focality was conducted on four representative montages. Focality was measured in MNI152 space as grey matter volume with an E-field strength equal to or exceeding 75% of the 99.9th percentile of the E-field distribution, as in previous works^65,66^. We investigated the relationships between focality and our independent variables using both simple correlational analyses and partial Spearman’s rank-order correlations. These findings were largely not significant and are provided in Supplementary Materials Tables 8 and 9.

### Feature extraction

In addition to age and gender, our tES dosage estimation models used anatomical features such as head circumference, cephalic index, BMI, and inter-electrode distance. The CamCAN repository provided information on age, gender, height, and weight, which were used to calculate BMI. In 54 out of 418 participants, BMI was not calculable due to missing data. For these cases, the missing BMI values were imputed using the median value of 24.05 kg/m^2^ (IQR: 20.71-30.56). As head circumferences and cephalic indexes were not included, we estimated these values using MRI scans.

Head circumference was measured by placing 60 points around the transverse plane slice of the MNI152 template; a similar approach was used by Antonenko et al.^17^. The inverse of the participant-specific affine linear transform was then applied to these points, and the sum of the Euclidean distance between the points in native space was used to estimate the head circumference. We compared our measured head circumference values to those predicted by Bushby et al.’s models^67^ to validate our approach. This model predicts mean head circumferences of 577mm for males and 553mm for females based on the height of participants in the dataset. This is within 6mm of our approach for males (mean±SD, 583mm±14.5mm) and 1mm of our approach for females (mean±SD, 552 ± 13.5 mm). This suggests that measuring head circumference with a tape measure is comparable to our MRI-based approach.

Head length and width were measured on the same transverse plane slice of the MNI152 template used for head circumference calculation. The length was measured as the distance between the most distal points of the forehead and the back of the head (mean ± SD, 190 mm ± 9.0 mm). The width was measured as the maximum distance between the most lateral points of the head along the axis perpendicular to the length axis (mean ± SD, 158 mm ± 6.7 mm). These measurements were transformed from MNI152 space to native space using the inverse of the linear affine transformation, as with the head circumference calculation. The cephalic index (mean ± SD, 83.1 ± 4.09) is the ratio of the head width to length, calculated as the head width divided by the length and multiplied by 100.

Inter-electrode distances were extracted in each participant’s native space after head models were re-oriented to a common right, anterior, superior (RAS) coordinate system. The inter-electrode distance was calculated as the Euclidean distance between the midpoints of the electrode meshes of the stimulation and return electrodes. In conventional montages, the inter-electrode distance was measured between the single return and stimulation electrodes. For HD montages, the inter-electrode distance was the average distance between the stimulation electrode and the four return electrodes.

**Fig. 8:**
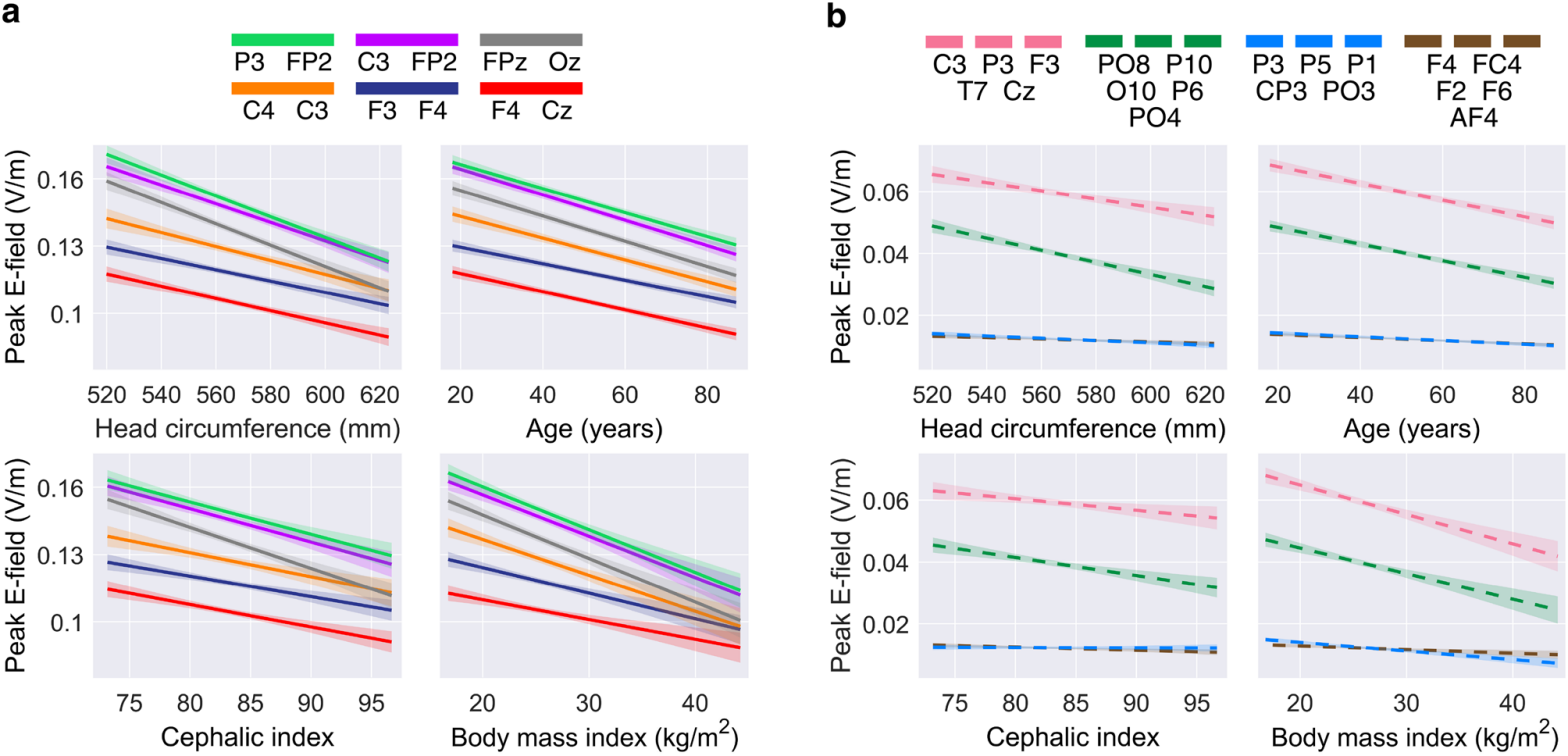
The relationships between demographic and morphological information and the peak E-field strength generated in the brain across a range of transcranial electrical stimulation (tES) montages. Each line represents an ordinary least squared linear regression, with the shaded area indicating the 95% confidence interval. These relationships are shown across **(a)** conventional montages and **(b)** high-definition (HD) montages.

Finally, data standardization was applied across all data types with appropriate techniques depending on their approximate distributions. Min-max scaling was applied to the uniformly distributed age, and z-score standardization was applied to the normally distributed head circumference, cephalic index, and peak E-field strength. Median interquartile range standardization was applied to the skewed BMI distribution, and gender values were set to 1 (male) or 0 (female). The relationships between these features and the peak E-field strength is shown in Fig. 8.

### Developing accessible models for the estimation of peak electric field strength

#### Montage-specific peak electric field strength estimation

To train our models to estimate peak E-field strength, we first compiled our testing and training datasets using MRI-informed simulations. We then analyzed the relationship between the features extracted and the peak E-field strengths using simple correlation analyses. Pearson’s linear correlation was applied to compare peak E-field strengths against head circumferences and cephalic indices, as these variables were normally distributed. For the non-normally distributed variables, age and BMI, we used Spearman’s Rho correlation. Additionally, we performed two-sample t-tests to compare the peak E-field strengths between genders. To understand the independent correlations of each variable, we conducted Spearman’s rank-order partial correlations. Bonferroni correction was applied to all tests to adjust for multiple comparisons.

After which, we trained a series of robust MLR models using an iteratively reweighted least squares approach with the bisquare weight function (tuning constant k=4.685). Multicollinearity was assessed across all MLR models using the variance inflation factor where values below 2.5 were considered acceptable. These models were designed to predict peak E-field strength values specific to each montage. The mathematical representation of these montage-specific models is shown in Eq. (1). The predicted montage-specific (MS) peak E-field strength is defined as $E_{MS}$. $\beta$ weights are present for each predictor, including age ($x_{age}$), gender ($x_{gender}$), head circumference ($x_{HC}$), cephalic index ($x_{CI}$), and BMI ($x_{BMI}$) as well as the intercept ($\beta _{MS_0}$).

To assess the accuracy of predicted peak E-field strengths ($E_{MS}$) compared to MRI-informed peak E-field strengths when 1mA of stimulation was applied, we employed 5-fold cross-validation. The model’s performance on each test set was evaluated using adjusted R^2^ and normalized root-mean-square error (NRMSE).

1 $$\begin{aligned} \begin{aligned} E_{\text {MS}} = \beta _{\text {MS}_1}x_{\text {age}} + \beta _{\text {MS}_2}x_{\text {HC}} + \beta _{\text {MS}_3}x_{\text {CI}} + \beta _{\text {MS}_4}x_{\text {BMI}} + \beta _{\text {MS}_5}x_{\text {gender}} + \beta _{\text {MS}_0}. \end{aligned} \end{aligned}$$

#### Montage-agnostic peak electric field estimation

Montage-agnostic models were designed to generalize to unseen electrode configurations, unlike montage-specific models, which are limited to the montage used in their training data. To achieve this, we trained separate montage-agnostic models on HD and conventional montages with 5-fold cross-validation across participants and leave-one-out cross validation across montages.

First, multiple linear regression models were trained for each montage in the training set as before. Next, regression coefficients ($\overline{\beta }$) were averaged across montages. Then, to incorporate inter-electrode distance which is a differentiating term between montages, E-field magnitudes were scaled ($S$) based on the electrode distance term ($x_{ED}$). This scaling with electrode distance was performed in a linear fashion as shown in Eq. 2.

2 $$\begin{aligned} \begin{aligned} E_{\text {LMA}} = {}&(S_{LMA}x_{\text {ED}}) \times (\overline{\beta }_{\text {LMA}_1}x_{\text {age}} + \overline{\beta }_{\text {LMA}_2}x_{\text {HC}} + \overline{\beta }_{\text {LMA}_3}x_{\text {CI}} + \overline{\beta }_{\text {LMA}_4}x_{\text {BMI}} + \overline{\beta }_{\text {LMA}_5}x_{\text {gender}} + \overline{\beta }_{\text {LMA}_6}x_{\text {ED}} + \overline{\beta }_{\text {LMA}_0}). \end{aligned} \end{aligned}$$

As well as a non-linear fashion as shown in Eq. 3 to capture the non-linear relationship between electrode distance and E-field strength^24^. As in montage-specific models, model performance on the respective test sets were evaluated using adjusted R^2^ and NRMSE.

3 $$\begin{aligned} \begin{aligned} E_{\text {NMA}} = {}&( S_{\text {NMA}_1} x_{ED}^{2} + S_{\text {NMA}_2} x_{ED} + S_{\text {NMA}_3}) \times (\overline{\beta }_{\text {NMA}_1}x_{\text {age}} + \overline{\beta }_{\text {NMA}_2}x_{\text {HC}} + \overline{\beta }_{\text {NMA}_3}x_{\text {CI}} + \overline{\beta }_{\text {NMA}_4}x_{\text {BMI}} + \overline{\beta }_{\text {NMA}_5}x_{\text {gender}} + \overline{\beta }_{\text {NMA}_0}). \end{aligned} \end{aligned}$$

#### Individualized tES dose estimation

Given the direct relationship between applied current and resulting E-field strength, the participant-specific current dosage, $I_\text {individualized}$, can be calculated using Eq. 4. The target peak E-field strength, $E_\text {target}$, can be set to any desired value depending on the specific requirements of a study or application. Traditionally, a current $I_\text {applied}$ was administered in MRI-informed participant-specific simulations to achieve a generated peak E-field strength, $E_\text {generated}$. The values of $E_\text {generated}$, $I_\text {applied}$, and $E_\text {target}$ were then used to determine the participant-specific current dosage, $I_\text {individualized}$.

4 $$\begin{aligned} \begin{aligned} I_{\text {individualized}} = \frac{I_{\text {applied}}}{ E_{\text {generated}}}\times E_{\text {target}}. \end{aligned} \end{aligned}$$

In our approach, the proposed models predict $E_\text {predicted}$ when 1 mA is applied (i.e., $I_\text {applied}$). Please note, in the results section, for illustration purposes, we set the target peak E-field strength as 0.1V/m. Levene’s tests with Bonferroni correction were applied to compare the variance of the resulting E-field distributions between standardized and fixed dosing.

## Supplementary Information

Supplementary Information.

## Data Availability

The regression model parameters generated in this study, along with Python and MATLAB code to apply these predictive models, are available in the following repository on GitHub: https://github.com/TothJake/tes-dose
