# Supplementary material for: Dose standardization for transcranial electrical stimulation: an accessible approach
Source: Sci Rep. 2025 Nov 25;15:41791. doi: 10.1038/s41598-025-25649-2 (PMC12647676; doi:10.1038/s41598-025-25649-2)
Supplement: Supplementary file 1 — Supplementary Information. [file 41598_2025_25649_MOESM1_ESM.docx]

**Supplementary Materials**


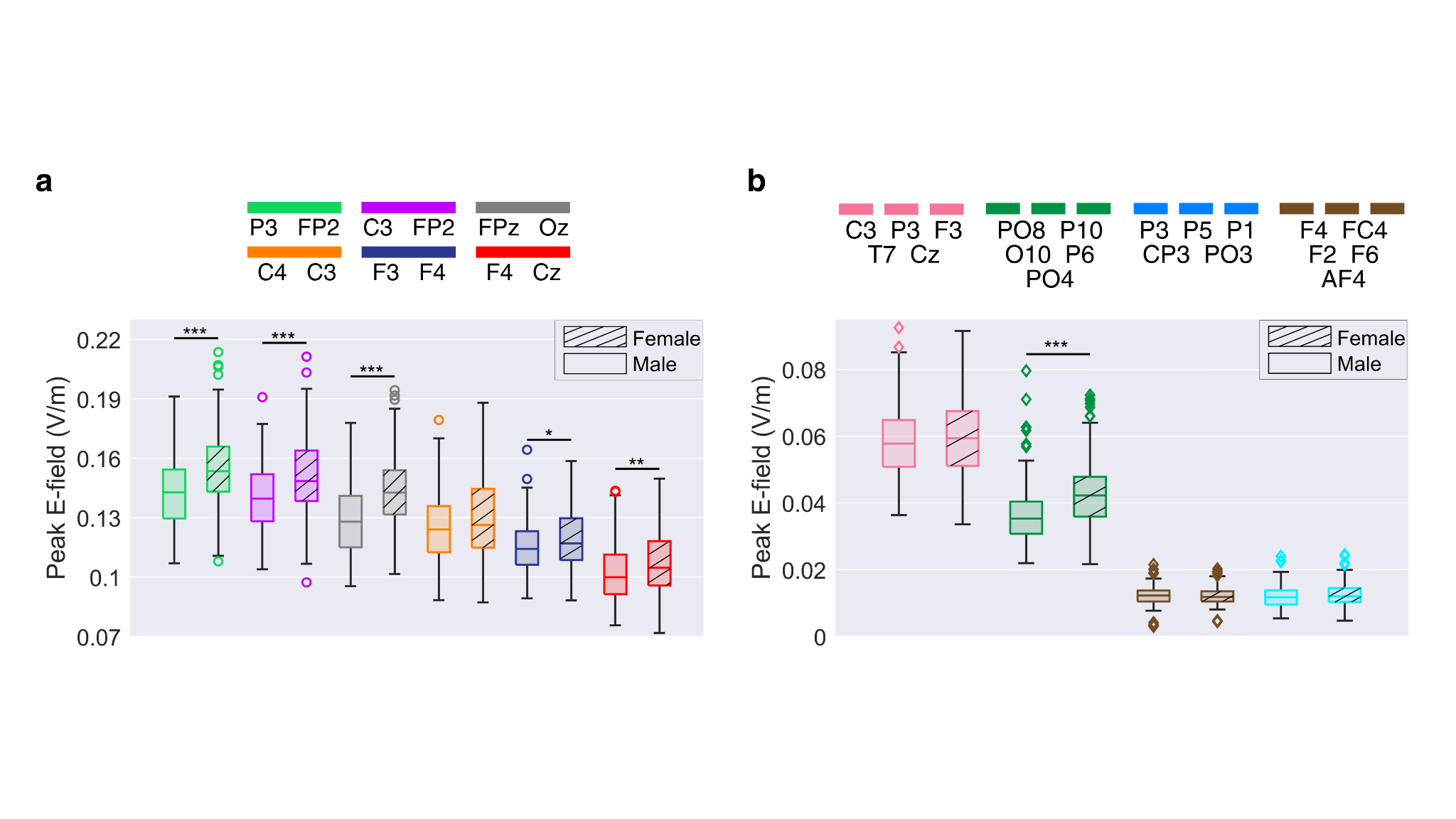


**Supplementary Figure 1.** Comparison of peak E-field strength between males and females. Peak E-fields were estimated from MRI-informed transcranial electrical stimulation (tES) modelling. A range of tES montages were simulated including (**a**) six conventional montages (anode:+1mA, cathode:-1mA) and (**b**) four high-definition montages (anode:+1mA, four cathodes each at -0.25mA). Statistical significance between genders was determined using Bonferroni-corrected two-sample t-tests. ***p<.001, **p<.01 and *p<.05. Box plots display the median (horizontal line), interquartile range (box), and data range (whiskers). Outliers are shown as circles in (a) and diamonds in (b).

| Montages | Pearson’s linear correlation | | | | Spearman’s Rho correlation | | | | Two-sample t-test |
| --- | --- | --- | --- | --- | --- | --- | --- | --- | --- |
|  | Head circumference | | Cephalic index | | Age | | BMI | | Gender |
|  | r | p | r | p | r | p | r | p | p |
| F4, Cz | -0.37 | <.001 | -0.28 | <.001 | -0.49 | <.001 | -0.26 | <.001 | <.05 |
| C3, FP2 | -0.45 | <.001 | -0.33 | <.001 | -0.57 | <.001 | -0.41 | <.001 | <.001 |
| F3, F4 | -0.35 | <.001 | -0.26 | <.001 | -0.46 | <.001 | -0.32 | <.001 | >0.1 |
| P3, FP2 | -0.51 | <.001 | -0.32 | <.001 | -0.55 | <.001 | -0.43 | <.001 | <.001 |
| C3, C4 | -0.33 | <.001 | -0.23 | <.001 | -0.48 | <.001 | -0.35 | <.001 | >0.1 |
| FPz, Oz | -0.51 | <.001 | -0.4 | <.001 | -0.56 | <.001 | -0.42 | <.001 | <.001 |
| F4, F2, AF4, F6, FC4 | -0.19 | <.01 | -0.13 | >0.1 | -0.39 | <.001 | -0.19 | <.05 | >0.1 |
| C3, P3, Cz, T7, F3 | -0.2 | <.01 | -0.12 | >0.1 | -0.42 | <.001 | -0.33 | <.001 | >0.1 |
| P3, P1, CP3, P5, PO3 | -0.2 | <.01 | -0.083 | >0.1 | -0.37 | <.001 | -0.33 | <.001 | >0.1 |
| PO8, P6, PO4, P10, O10 | -0.4 | <.001 | -0.24 | <.001 | -0.5 | <.001 | -0.36 | <.001 | <.001 |

**Supplementary Table 1.** Correlational analyses between each independent variable and the MRI-informed peak E-field strengths across all montages. Bonferroni correction was applied to account for multiple comparisons.

| Conventional montages | Adjusted R2 | | | | | | NRMSE | | | | | |
| --- | --- | --- | --- | --- | --- | --- | --- | --- | --- | --- | --- | --- |
|  | Fold 1 | Fold 2 | Fold 3 | Fold 4 | Fold 5 | Average | Fold 1 | Fold 2 | Fold 3 | Fold 4 | Fold 5 | Average |
| F4, Cz | 0.456 | 0.369 | 0.275 | 0.243 | 0.381 | 0.345 | 0.102 | 0.110 | 0.119 | 0.116 | 0.105 | 0.110 |
| C3, FP2 | 0.548 | 0.581 | 0.524 | 0.468 | 0.396 | 0.503 | 0.083 | 0.080 | 0.086 | 0.085 | 0.091 | 0.085 |
| F3, F4 | 0.312 | 0.248 | 0.331 | 0.421 | 0.184 | 0.299 | 0.100 | 0.106 | 0.095 | 0.089 | 0.107 | 0.099 |
| P3, FP2 | 0.627 | 0.548 | 0.526 | 0.448 | 0.429 | 0.516 | 0.074 | 0.081 | 0.084 | 0.085 | 0.087 | 0.082 |
| C3, C4 | 0.141 | 0.409 | 0.323 | 0.356 | 0.279 | 0.302 | 0.133 | 0.111 | 0.120 | 0.112 | 0.119 | 0.119 |
| FPz, Oz | 0.546 | 0.616 | 0.593 | 0.597 | 0.574 | 0.585 | 0.090 | 0.083 | 0.086 | 0.082 | 0.084 | 0.085 |

**Supplementary Table 2.** Montage-specific model performance for conventional montages across each training fold in terms of adjusted R² and normalised root mean squared error (NRMSE). Higher adjusted R² and lower NRMSE values indicate a better model fit.

| Conventional montages | Adjusted R2 | | | | | | NRMSE | | | | | |
| --- | --- | --- | --- | --- | --- | --- | --- | --- | --- | --- | --- | --- |
|  | Fold 1 | Fold 2 | Fold 3 | Fold 4 | Fold 5 | Average | Fold 1 | Fold 2 | Fold 3 | Fold 4 | Fold 5 | Average |
| F4, Cz | 0.323 | 0.368 | 0.245 | 0.316 | 0.306 | 0.312 | 0.111 | 0.110 | 0.124 | 0.109 | 0.107 | 0.112 |
| C3, FP2 | 0.470 | 0.487 | 0.386 | 0.501 | 0.417 | 0.452 | 0.085 | 0.092 | 0.101 | 0.076 | 0.082 | 0.087 |
| F3, F4 | 0.332 | 0.256 | 0.203 | 0.113 | 0.257 | 0.232 | 0.099 | 0.104 | 0.103 | 0.104 | 0.102 | 0.102 |
| P3, FP2 | 0.436 | 0.556 | 0.423 | 0.470 | 0.392 | 0.455 | 0.087 | 0.080 | 0.096 | 0.086 | 0.081 | 0.086 |
| C3, C4 | 0.357 | 0.165 | 0.189 | 0.259 | 0.060 | 0.206 | 0.111 | 0.120 | 0.140 | 0.121 | 0.130 | 0.124 |
| FPz, Oz | 0.441 | 0.540 | 0.419 | 0.492 | 0.381 | 0.454 | 0.101 | 0.091 | 0.104 | 0.091 | 0.083 | 0.094 |

**Supplementary Table 3.** Montage-agnostic linear model performance for conventional montages across each training fold in terms of adjusted R² and normalised root mean squared error (NRMSE). Higher adjusted R² and lower NRMSE values indicate a better model fit.

| Conventional montages | Adjusted R2 | | | | | | NRMSE | | | | | |
| --- | --- | --- | --- | --- | --- | --- | --- | --- | --- | --- | --- | --- |
|  | Fold 1 | Fold 2 | Fold 3 | Fold 4 | Fold 5 | Average | Fold 1 | Fold 2 | Fold 3 | Fold 4 | Fold 5 | Average |
| F4, Cz | 0.350 | 0.296 | 0.219 | 0.277 | 0.235 | 0.275 | 0.109 | 0.116 | 0.126 | 0.112 | 0.113 | 0.115 |
| C3, FP2 | 0.515 | 0.486 | 0.406 | 0.519 | 0.393 | 0.464 | 0.081 | 0.092 | 0.099 | 0.074 | 0.084 | 0.086 |
| F3, F4 | 0.364 | 0.211 | 0.219 | 0.093 | 0.247 | 0.227 | 0.096 | 0.107 | 0.102 | 0.105 | 0.103 | 0.103 |
| P3, FP2 | 0.470 | 0.577 | 0.433 | 0.497 | 0.390 | 0.473 | 0.084 | 0.078 | 0.095 | 0.084 | 0.081 | 0.084 |
| C3, C4 | 0.401 | 0.095 | 0.210 | 0.314 | 0.039 | 0.212 | 0.107 | 0.125 | 0.138 | 0.116 | 0.132 | 0.124 |
| FPz, Oz | 0.476 | 0.574 | 0.444 | 0.527 | 0.400 | 0.484 | 0.097 | 0.088 | 0.102 | 0.088 | 0.082 | 0.091 |

**Supplementary Table 4.** Montage-agnostic non-linear model performance for conventional montages across each training fold in terms of adjusted R² and normalised root mean squared error (NRMSE). Higher adjusted R² and lower NRMSE values indicate a better model fit.

| HD  montages | Adjusted R2 | | | | | | NRMSE | | | | | |
| --- | --- | --- | --- | --- | --- | --- | --- | --- | --- | --- | --- | --- |
|  | Fold 1 | Fold 2 | Fold 3 | Fold 4 | Fold 5 | Average | Fold 1 | Fold 2 | Fold 3 | Fold 4 | Fold 5 | Average |
| F4, F2, AF4, F6, FC4 | 0.090 | 0.110 | 0.104 | 0.005 | 0.265 | 0.115 | 0.210 | 0.210 | 0.191 | 0.201 | 0.175 | 0.197 |
| C3, P3, Cz, T7, F3 | 0.271 | 0.160 | 0.180 | 0.299 | 0.195 | 0.221 | 0.159 | 0.174 | 0.162 | 0.149 | 0.161 | 0.161 |
| P3, P1, CP3, P5, PO3 | 0.184 | 0.076 | 0.273 | 0.194 | -0.003 | 0.145 | 0.250 | 0.266 | 0.222 | 0.237 | 0.268 | 0.249 |
| PO8, P6, PO4, P10, O10 | 0.245 | 0.381 | 0.467 | 0.330 | 0.365 | 0.358 | 0.208 | 0.188 | 0.177 | 0.205 | 0.185 | 0.193 |

**Supplementary Table 5.** Montage-specific model performance for high definition (HD) montages across each training fold in terms of adjusted R² and normalised root mean squared error (NRMSE). Higher adjusted R² and lower NRMSE values indicate a better model fit.

| HD montages | Adjusted R2 | | | | | | NRMSE | | | | | |
| --- | --- | --- | --- | --- | --- | --- | --- | --- | --- | --- | --- | --- |
|  | Fold 1 | Fold 2 | Fold 3 | Fold 4 | Fold 5 | Average | Fold 1 | Fold 2 | Fold 3 | Fold 4 | Fold 5 | Average |
| F4, F2, AF4, F6, FC4 | 0.070 | -0.027 | -0.083 | 0.031 | 0.002 | -0.001 | 0.210 | 0.225 | 0.209 | 0.210 | 0.180 | 0.207 |
| C3, P3, Cz, T7, F3 | 0.156 | 0.245 | 0.162 | 0.176 | 0.137 | 0.175 | 0.143 | 0.155 | 0.189 | 0.172 | 0.163 | 0.164 |
| P3, P1, CP3, P5, PO3 | 0.188 | 0.077 | 0.065 | 0.057 | 0.105 | 0.098 | 0.217 | 0.254 | 0.276 | 0.289 | 0.247 | 0.256 |
| PO8, P6, PO4, P10, O10 | 0.159 | 0.473 | 0.202 | 0.264 | 0.189 | 0.257 | 0.232 | 0.153 | 0.232 | 0.197 | 0.195 | 0.202 |

**Supplementary Table 6.** Montage-agnostic linear model performance for high definition (HD) montages across each training fold in terms of adjusted R² and normalised root mean squared error (NRMSE). Higher adjusted R² and lower NRMSE values indicate a better model fit.

| HD montages | Adjusted R2 | | | | | | NRMSE | | | | | |
| --- | --- | --- | --- | --- | --- | --- | --- | --- | --- | --- | --- | --- |
|  | Fold 1 | Fold 2 | Fold 3 | Fold 4 | Fold 5 | Average | Fold 1 | Fold 2 | Fold 3 | Fold 4 | Fold 5 | Average |
| F4, F2, AF4, F6, FC4 | 0.070 | -0.029 | -0.084 | -0.366 | 0.002 | -0.081 | 0.210 | 0.225 | 0.209 | 0.249 | 0.180 | 0.215 |
| C3, P3, Cz, T7, F3 | 0.163 | 0.255 | 0.169 | 0.170 | 0.148 | 0.181 | 0.142 | 0.154 | 0.188 | 0.172 | 0.161 | 0.164 |
| P3, P1, CP3, P5, PO3 | 0.178 | 0.070 | 0.073 | 0.059 | 0.095 | 0.095 | 0.219 | 0.255 | 0.274 | 0.289 | 0.248 | 0.257 |
| PO8, P6, PO4, P10, O10 | 0.159 | 0.469 | 0.204 | 0.265 | 0.185 | 0.256 | 0.232 | 0.154 | 0.232 | 0.197 | 0.196 | 0.202 |

**Supplementary Table 7.** Montage-agnostic non-linear model performance for high definition (H) montages across each training fold in terms of adjusted R² and normalised root mean squared error (NRMSE). Higher adjusted R² and lower NRMSE values indicate a better model fit.

| Montages | Pearson’s linear correlation | | | | Spearman’s Rho correlation | | | | Two-sample t-test |
| --- | --- | --- | --- | --- | --- | --- | --- | --- | --- |
|  | Head circumference | | Cephalic index | | Age | | BMI | | Gender |
|  | r | p | r | p | r | p | r | p | p |
| F4, Cz | -0.082 | >0.1 | -0.32 | <.001 | -0.51 | <.001 | 0.076 | >0.1 | <.05 |
| FPz, Oz | -0.23 | <.001 | -0.13 | >0.1 | -0.41 | <.001 | -0.017 | >0.1 | <.001 |
| F4, F2, AF4, F6, FC4 | -0.018 | >0.1 | 0.16 | <.05 | -0.1 | >0.1 | 0.15 | <.05 | >0.1 |
| C3, P3, Cz, T7, F3 | 0.15 | >0.1 | 0.19 | <.01 | 0.007 | >0.1 | 0.11 | =0.55 | >0.1 |

**Supplementary Table 8.** Simple correlational analyses for each independent variable and the MRI-informed E-field focality across four representative montages. Bonferroni correction was applied to account for multiple comparisons.

| Montages | Partial Spearman’s rank-order correlations | | | | | | | | | |
| --- | --- | --- | --- | --- | --- | --- | --- | --- | --- | --- |
|  | Head circumference | | Cephalic index | | Age | | BMI | | Gender | |
|  | r | p | r | p | r | p | r | p | r | p |
| F4, Cz | -0.037 | >0.1 | -0.024 | >0.1 | -0.22 | <.001 | 0.13 | >0.1 | -0.14 | >0.1 |
| FPz, Oz | -0.049 | >0.1 | 0.051 | >0.1 | -0.47 | <.001 | 0.1 | >0.1 | 0.15 | <.05 |
| F4, F2, AF4, F6, FC4 | -0.034 | >0.1 | -0.18 | <.01 | -0.51 | <.001 | 0.12 | >0.1 | 0.081 | >0.1 |
| C3, P3, Cz, T7, F3 | 0.044 | >0.1 | 0.25 | <.001 | -0.24 | <.001 | 0.15 | <.05 | 0.076 | >0.1 |

**Supplementary Table 9.** Partial Spearman’s rank-order correlational analyses for each independent variable independent of all others against the MRI-informed E-field focality across four representative montages. Bonferroni correction was applied to account for multiple comparisons.
